# Improving protein fold recognition using the amalgamation of evolutionary-based and structural based information

**DOI:** 10.1186/1471-2105-15-S16-S12

**Published:** 2014-12-08

**Authors:** Kuldip K Paliwal, Alok Sharma, James Lyons, Abdollah Dehzangi

**Affiliations:** 1 School of Engineering, Griffith University, Brisbane, Australia; 2 School of Engineering and Physics, University of the South Pacific, Suva, Fiji; 3 Institute for Integrated and Intelligent Systems (IIIS), Brisbane, Australia; 4 National ICT Australia (NICTA), Brisbane, Australia

## Abstract

Deciphering three dimensional structure of a protein sequence is a challenging task in biological science. Protein fold recognition and protein secondary structure prediction are transitional steps in identifying the three dimensional structure of a protein. For protein fold recognition, evolutionary-based information of amino acid sequences from the position specific scoring matrix (PSSM) has been recently applied with improved results. On the other hand, the SPINE-X predictor has been developed and applied for protein secondary structure prediction. Several reported methods for protein fold recognition have only limited accuracy. In this paper, we have developed a strategy of combining evolutionary-based information (from PSSM) and predicted secondary structure using SPINE-X to improve protein fold recognition. The strategy is based on finding the probabilities of amino acid pairs (AAP). The proposed method has been tested on several protein benchmark datasets and an improvement of 8.9% recognition accuracy has been achieved. We have achieved, for the first time over 90% and 75% prediction accuracies for sequence similarity values below 40% and 25%, respectively. We also obtain 90.6% and 77.0% prediction accuracies, respectively, for the Extended Ding and Dubchak and Taguchi and Gromiha benchmark protein fold recognition datasets widely used for in the literature.

## Introduction

Recognition of protein folds is an essential step in identifying the tertiary structure of proteins. The identification of protein tertiary structures helps in analysing and understanding function, heterogeneity and protein-protein and protein-peptide interactions. The protein fold recognition problem can be tackled by first extracting useful and informative features from protein sequences followed by the identification of the fold of a novel protein sequence using an appropriate classifier. A range of techniques have been developed addressing both the feature extraction and classification areas. Protein fold recognition comprises two major steps: feature extraction and classification.

For feature extraction, several techniques, based on structural, physicochemical and evolutionary information, are available. Dubchak et al. [1] have shown importance of syntactical and physicochemical features in protein fold recognition using amino acid composition (AAC), in conjunction with five physicochemical attributes of amino acids: hydrophobicity (H), polarity (P), van der Waals volume (V), predicted secondary structure based on normalized frequency of  α-helix (X) and polarizability (Z). Their 120-dimensional feature set is composed of 20 AAC together with 105 physicochemical features. Their features have been extensively used in protein fold recognition [2-13]. There are other attributes used to extract features after [1]. These are size of the amino acid side chain [9], solvent accessibility [14], flexibility [15], bulkiness [16], first and second order entropy [17]. As the selection of these attributes was done arbitrarily, we have proposed a more systematic approach to attribute selection has been proposed [18,19]. Further, a profile-profile alignment method is proposed by Ohlson et al., [20] to improve protein fold recognition. The syntactical-based features using amino acid occurrence are proposed by [21] and by using amino acid residues along with residue pairs are proposed in [22]. In [23], authors have proposed pairwise frequencies in two ways: PF1 for amino acids separated by one residue and PF2 for adjacent amino acid residues, where PF1 and PF2 are 400-dimensional each. These features are further concatenated in [24] resulting in 800 features. In some cases, the dimensionality of features could be large which increases computational complexity of the classifier used. In this case, feature selection methods can be used as a preprocessing step to reduce the number of feature [25-27]. To present protein sequence in an effective manner, authors in [28] proposed pseudo-amino acid composition (A) features. In [29], authors proposed autocross-covariance (ACC) transformation and the work in [30-32] has shown protein sequence autocorrelation. In [9], authors derived additional features from physicochemical properties. The bi-gram features [19] using evolutionary based information (PSSM) have also shown effective recognition results. For more feature extraction or selection methods please see [33-40].

For the classification step, a variety of algorithms, such as linear discriminant analysis [41], Bayesian classifiers [2], Bayesian decision rule [42], k-nearest neighbor [30], Hidden Markov model [43,44], artificial neural network [45,46], support vector machine (SVM) [5,22,23] and ensemble classifiers [6,24,47,48], have been adopted. Among the various protein fold recognition classifiers reported in the literature, SVM (or SVM-based) classifiers demonstrate excellent performance [23,31,32].

Since the feature extraction is crucial in protein structure recognition, our approach is focussed on developing an appropriate feature extraction method. There can be four distinct types of features, extracted from protein sequence: sequential-based, physicochemical-based, structural-based and evolutionary-based features. In this work, we have investigated evolutionary-based and structural-based features perform as done by other authors [24,29].

The evolutionary information is extracted from PSSM matrices (a publically available tool to retrieve the PSSM matrix is PSI-BLAST) [49]. PSSM matrix estimates the relative probability of amino acid substitution. If a protein sequence is of length *L *then PSSM matrix would have *L *rows and 20 columns (since there are maximum of 20 distinct amino acids in a protein sequence). The structural information is extracted from predicted secondary structure of the proteins using predictors such as SPINE-X and PSIPRED [50,51]. Protein secondary structures are classified into three states namely, alpha-helix, beta-strands and coils. Since SPINE-X outperformed PSIPRED for protein secondary structure prediction [50], we use SPINE-X in this study. For a protein sequence of length *L*, SPINE-X provides a matrix of probabilities of size *L *× 3 (where 3 refers to the number of secondary structure states). This matrix contains useful information for secondary structure class prediction.

In this paper, we combine the information from the PSSM matrix and secondary structure prediction matrix (SSPM) from SPINE-X to extract relevant and useful knowledge for protein fold recognition. The motivation of combining these two categories comes from the fact that they produce high performance in fold recognition and secondary structure prediction, respectively. Therefore, they have extracted relevant information for the respective tasks and if their impact can be utilized as a whole then the performance of fold recognition can be appreciated. Considering this, we developed *k*-amino acid pair (AAP) feature extraction method based on PSSM and SSPM, and show its usefulness on several protein benchmark datasets. Compared to the best results reported in the literature, we have enhanced the recognition accuracy by 8.9% and 4.7% for sequence similarity values of less than 25% and 40%, respectively. The next section covers materials and methods.

## Materials and Methods

### The *k*-AAP feature extraction method

In this section, we describe *k*-AAP method using PSSM linear probabilities and SSPM (from SPINE X). PSSM is calculated by applying PSIBLAST [49] in which its cut off value (E) is set to 0.001 on our explored benchmarks (using NCBI's non redundant protein database). Here, we represent a protein sequence by its PSSM and SSPM and compute ordered pair features of amino acids using the probability information from PSSM and SSPM. Let *P *be the matrix of PSSM linear probabilities and *S *be the matrix for SSPM values. For an *L *length protein sequence, the size of *P *would be L×20 and the size of *S *would be *L*×6. If we denote matrix *Q *as Q=[P,S] then its size would be L×23. The matrix *Q *contains probability information of amino acids and secondary structure component corresponding to each amino acid in a protein sequence. The *i*th-row and *j*th-column element, $q_{i,j}$ of *Q *is the probability of an amino acid or secondary structure. The information in *Q *is shown to be useful by gathering amino acid pairs in the following manner:

(1) $$R_{k}\left(m,n\right)=\overset{L-k}{\underset{i=1}{\sum }}q_{i,m}q_{i+k,n},\;\text{where }1\leq m\leq 23\;\text{and}\;1\leq n\leq 23$$

Equation 1 will give 23×23 pairwise probabilities of $R_{k}\left(m,n\right)$. It can be interpreted in the form of feature vector of dimension 529 as:

(2) $$f\left(R_{k}\right)=\left[R_{k}\left(1,1\right),R_{k}\left(1,2\right),\ldots ,R_{k}\left(1,23\right),R_{k}\left(2,1\right),\ldots ,R_{k}\left(23,1\right),\ldots ,R_{k}\left(23,23\right)\right]$$

In this work, we used k=1,2,3 and 4. We observed that by using higher values of *k*, the performance does not improve further. This is because as we increase *k *the correlation between the two amino acids decreases which do not provide relevant information for fold recognition. By using the representation of the above feature vector $f\left(R_{k}\right)$ for all values of *k*, we can denote feature vector *F *as $F={\left[f\left(R_{1}\right),f\left(R_{2}\right),f\left(R_{3}\right),f\left(R_{4}\right)\right]}^{T}$, where superscript T is the transpose of the vector. The dimensionality of this feature vector would be 2116. From the feature vector computation, we note that all PSSM and SSPM probability information have been utilized. From a biological perspective, proteins with the same fold also share similar general secondary structure information. In other words, proteins with the same fold often have highly conserved amino acid sub-sequences and can translate to a specific secondary structure residue. In these conserved regions, *k *-AAP probability values effectively characterize the amino acid sub-sequences. For each sub-sequence conserved in a fold and/or related to a particular residue, all proteins with that fold will contain amino acid pairs characterizing that conserved region and/or set of residues. This information can therefore filter out folds that do not share the same amino acid sub-sequences. Therefore, intuitively *F *contains more useful information for fold recognition. This has been demonstrated in the experimentation part of the paper.

For classification of the feature vectors, we used the support vector machine (SVM) classifier as it has shown promising results in protein fold recognition. We employed SVM from libsvm with RBF kernel [52]. The parameters of SVM are optimized by using grid search.

**Figure 1:**
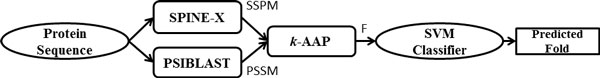
**An overview of *k*-AAP feature extraction scheme**.

In order to present an overview of the proposed strategy, we show a flow diagram of the proposed method in Figure 1. The input (in the figure) is a protein sequence and the output is recognized protein fold.

### Support Vector Machine as a classifier

SVM [53] is used as a classifier in this experiment. It is one of the leading classification technique and has also been applied in regression areas. The goal of SVM is to discover maximum margin hyper plane (MMH) in order to reduce misclassification error. Data in SVM is transformed through a kernel *K *function (e.g. linear or RBF) [54,55].

The SVM classifier attempts to find separation between two classes. If the class label of an input space vector $x_{i}$ is $y_{i}$, where $y_{i}$ is either -1 or +1. Then any unknown vector $x^{\prime }$ would have class label

(3) $$y^{\prime }=\text{sign}\left(\overset{n}{\underset{i=1}{\sum }}\alpha_{i}y_{i}K\left(x_{i},x^{\prime }\right)+b\right)$$

where $y^{\prime }$ denotes the predicted class label of $x^{\prime }$; K(.,.) is kernel function; number of support vectors is defined by *n*; bias is defined by b and adjustable weights are defined by $\alpha_{i}$. In this work, LibSVM [52] has been used to conduct training and testing of data. The kernel function utilized is radial basis function (RBF) which is defined by $K\left(z_{i},z_{j}\right)=\text{exp}\left(-g*||z_{i}-z_{j}|{|}^{2}\right)$, where *g *is gamma parameter. The gamma and complexity parameter (*C*) parameters are optimized using LibSVM. The data is not normalized before processing to the SVM classifier.

### Dataset

We have used three protein sequence datasets in this study: 1) Ding and Dubchak (DD) [5], 2) Taguchi and Gromiha (TG) [21] and 3) Extended DD (EDD) [29]. The DD-dataset utilizes protein sequences from 27 Structural Classification of Proteins (SCOP) folds comprehensively, comprehensively covering  α,  β, α/β and α+β structural classes [5]. The training set contains 311 protein sequences with no two proteins having more than 35% of sequence identity for alignments longer than 80 residues. The test set comprises 383 protein sequences of less than 40% sequence identity. The training and test sets were merged for analysis.

TG-dataset has 30 folds of globular proteins from SCOP. It has a total of 1612 protein sequences with sequence similarity no more than 25%. The dataset has been described in detail in Taguchi and Gromiha [21].

EDD-dataset comprises 27 folds which are also present in the DD-dataset. This dataset has 3418 proteins with sequence similarity less than 40%. In this study, we have used the approach described by Dong et al. [29] to extract the EDD-dataset from SCOP.

We perform *n*-fold cross-validation process, where *n *= 5, 6, 7, 8, 9 and 10 for analysis and observation. The next section describes the experimental part of the work.

## Results and Discussion

The proposed *k*-AAP features have been compared with PF1, PF2 [23], PF [24], Occurrence [21], AAC and AAC+HXPZV [5] feature extraction methods. Moreover, we have updated the protein sequences to get the consensus sequence by using their corresponding PSSMs; i.e., each amino acid of a protein sequence is replaced by the amino acid that has the highest probability in PSSM. After this updating procedure, we have used the same feature extraction techniques (PF1, PF2, PF, O, AAC and AAC+HXPZV) again to obtain the recognition performance. In Tables 1, 2, 3, we have placed the results for PSSM updated protein sequences (or the consensus sequence) in the columns 2-7 of the row of PSSM + FET, where FET is any feature extraction technique. We have also used PSSM based mono-gram and bi-gram feature extraction methods [19] and ACC [29] for comparison and the highest recognition accuracy of each *n*-fold cross-validation is highlighted in bold face.

**Table 1:** Recognition accuracy by *n*-fold cross validation procedure for different feature extraction techniques for SVM classification for the DD-dataset.

Feature sets	*n *= 5	*n *= 6	*n *= 7	*n *= 8	*n *= 9	*n *= 10
PF1 [23]	48.6	49.1	49.5	50.1	50.5	50.6
PF2 [23]	46.3	47.0	47.5	47.7	47.9	48.2
PF [24]	51.2	52.2	52.6	52.9	53.4	53.4
O [21]	49.7	50.4	50.8	50.8	51.1	51.0
AAC [5]	43.6	43.9	44.2	44.8	44.6	45.1
AAC+HXPZV [5]	45.1	46.2	46.5	46.8	46.9	47.2
ACC [29]	65.7	66.6	66.8	67.5	67.7	68.0
PSSM+PF1 [55]	62.5	63.2	63.7	64.2	64.5	64.6
PSSM+PF2 [55]	62.7	63.3	64.1	64.2	64.6	64.7
PSSM+PF [55]	65.5	66.2	66.5	66.9	67.1	67.5
PSSM+O [55]	62.5	62.1	62.5	62.9	63.4	63.5
PSSM+AAC [55]	57.5	58.1	58.4	58.7	59.1	59.2
PSSM+AAC+HXPZV [55]	55.9	56.9	57.1	57.7	58.0	58.2
Mono-gram [19]	67.7	68.4	68.6	69.1	69.4	69.6
Bi-gram [19]	72.6	73.1	73.7	73.7	74.1	74.1
*k*-AAP (this paper)	**74.3**	**75.2**	**75.2**	**75.7**	**76.1**	**76.1**

**Table 2:** Recognition accuracy by *n*-fold cross validation procedure for different feature extraction techniques for SVM classification for the TG dataset.

Feature sets	*n *= 5	*n *= 6	*n *= 7	*n *= 8	*n *= 9	*n *= 10
PF1 [23]	38.1	38.4	38.6	38.7	38.8	38.8
PF2 [23]	38.0	38.4	38.5	38.6	38.7	38.8
PF [24]	42.3	42.6	42.7	43.0	43.0	43.1
O [21]	35.8	36.1	36.2	36.1	36.3	36.3
AAC [5]	31.5	31.5	31.7	31.8	31.9	32.0
AAC+HXPZV [5]	35.7	36.0	36.1	36.2	36.3	36.3
ACC [29]	64.9	65.4	65.9	66.2	66.4	66.4
PSSM+PF1 [55]	51.1	51.5	52.0	52.3	52.4	52.7
PSSM+PF2 [55]	50.2	50.4	50.7	50.8	51.0	51.1
PSSM+PF [55]	57.2	57.8	58.0	58.3	58.5	58.8
PSSM+O [55]	46.0	46.3	46.5	46.5	46.7	46.7
PSSM+AAC [55]	43.2	43.5	43.6	43.8	43.8	44.0
PSSM+AAC+HXPZV [55]	45.6	45.9	46.0	46.2	46.3	46.6
Mono-gram [19]	57.2	57.3	58.2	58.4	58.8	58.8
Bi-gram [19]	67.1	67.5	67.6	67.8	68.1	68.1
*k *-AAP (this paper)	**75.9**	**76.2**	**76.6**	**76.7**	**76.9**	**77.0**

**Table 3:** Recognition accuracy by *n*-fold cross validation procedure for different feature extraction techniques for SVM classification for the EDD dataset.

Feature sets	*n *= 5	*n *= 6	*n *= 7	*n *= 8	*n *= 9	*n *= 10
PF1 [23]	50.2	50.5	50.5	50.7	50.8	50.8
PF2 [23]	49.3	49.5	49.7	49.8	49.8	49.9
PF [24]	54.7	55.0	55.2	55.4	55.5	55.6
O [21]	46.4	46.6	46.6	46.7	46.7	46.9
AAC [5]	40.3	40.6	40.7	40.7	40.9	40.9
AAC+HXPZV [5]	40.2	40.4	40.6	40.7	40.9	40.9
ACC [29]	84.9	85.2	85.4	85.6	85.8	85.9
PSSM+PF1 [55]	74.1	74.5	74.7	75.0	75.1	75.2
PSSM+PF2 [55]	73.7	74.1	74.5	74.6	74.7	74.9
PSSM+PF [55]	78.2	78.6	78.8	79.0	79.1	79.3
PSSM+O [55]	67.6	68.0	68.1	68.3	68.3	68.5
PSSM+AAC [55]	60.9	61.3	61.5	61.6	61.7	61.9
PSSM+AAC+HXPZV [55]	66.7	67.2	67.4	67.7	67.8	67.9
Mono-gram [19]	76.2	76.3	76.6	76.8	77.0	76.9
Bi-gram [19]	83.6	84.0	84.1	84.3	84.3	84.5
*k *-AAP (this paper)	**90.1**	**90.2**	**90.4**	**90.5**	**90.6**	**90.6**

Table 1 shows that the highest accuracy obtained by *k*-AAP is 76.1% on DD-dataset which is at least 2% higher than the other techniques. On TG-dataset (Table 2), *k*-AAP achieved 77.0% accuracy which is around 10.6% better than Dong et al., [29] results and 8.9% better than the best results achieved for this benchmark [19]. It is important to highlight that this enhancement is achieved by using 2116 (4×529) features compared to 4000 features used in Dong et al., [29] study. For EDD-dataset (Table 3), *k *-AAP achieved 90.6% accuracy which is around 4.7% higher than the other techniques. This enhancement in prediction accuracy is obtained at low sequence similarity of proteins (less than 25% sequence similarity for TG dataset and less than 40% sequence similarity for EDD dataset). This shows that the extracted features are able to maintain their discriminatory information when the sequence similarity is reduced. Therefore, it can be deduced that *k*-AAP is performing quite well in recognizing protein folds.

To analyse the statistical significance of the prediction accuracy obtained for protein fold recognition, we carried out paired t-test on our results obtained from the experiments and the highest accuracies reported in the literature. Our results indicate an associated probability value of p=0.0015 from the paired t-test. This value confirms that the reported improvement in this work compared to the results found in the literature is significant

We have also carried out experiments to find out which terms either PSSM or SSPM contribute the most in protein fold recognition. In order to do this, we used k=4 and conducted 10-fold cross-validation using PSSM separately and SSPM separately. The results are shown in Table 4 indicating that accuracy is much higher using PSSM based features compared to SSPM based features, and that the accuracy achieved by their combination is the highest. Therefore, we can say that PSSM based features are more contributing towards the overall performance of *k *-AAP method. Nonetheless, SSPM is also playing important role in improving the performance further.

**Table 4:** Recognition accuracy (in percentage) for 10-fold cross validation procedure for PSSM and SSPM using SVM classifier on the DD, TG and EDD datasets.

Feature sets	DD	TG	EDD
Using PSSM only	74.5	73.8	88.8
Using SSPM only	59.8	55.2	71.7
Using PSSM+SSPM (i.e., *k *-AAP)	76.1	77.0	90.6

Moreover, we have shown our feature extraction method with other classifiers in order to demonstrate the significance of evolutionary and structural information. Table 5 depicts protein fold recognition accuracy on all the three datasets using 10-fold cross-validation. It can be seen that the results obtained by other classifiers are encouraging. This shows that evolutionary and structural information play a crucial role in extraction important discriminant information for protein fold recognition. At the same time, the accuracies obtained by other classifiers are slightly lower than the SVM classifier used. This confirms our selection of using SVM classifier for various feature extraction methods.

**Table 5:** Recognition accuracy (in percentage) for 10-fold cross validation procedure using different classifiers on *k *-AAP.

Classifiers	DD	TG	EDD
Naïve Bayes	62.3	48.5	58.2
SVM (SMO with linear polynomial of degree P = 1)	75.4	76.1	88.8
SVM (SMO with P = 3)	69.1	69.2	86.2
Random Forest (10 base learners)	62.9	52.1	73.0
Adaboost.M1 (10 base learners)	68.1	59.3	79.2
kNN (for *k *= 1)	70.8	65.6	84.3

**Figure 2:**
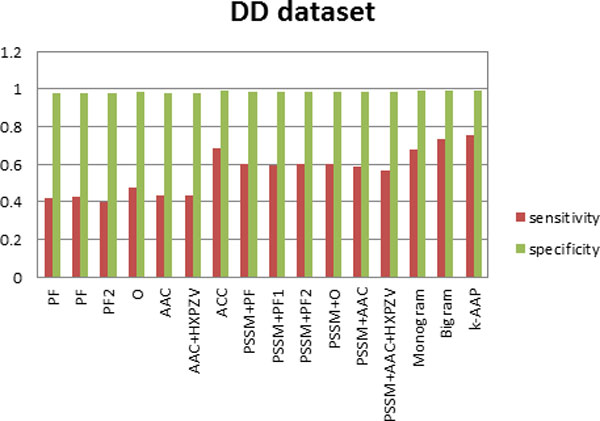
**Sensitivity and specificity of all feature sets for the DD dataset**.

**Figure 3:**
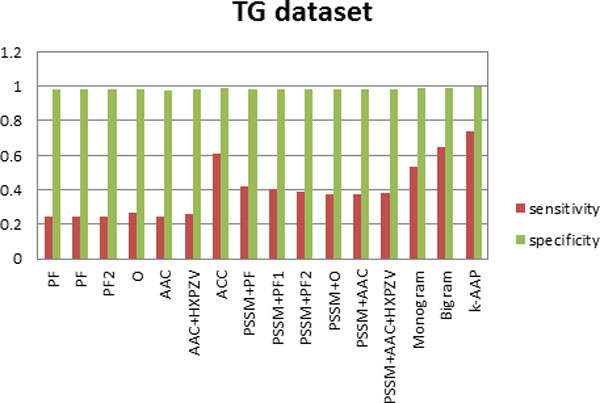
**Sensitivity and specificity of all feature sets for the TG dataset**.

**Figure 4:**
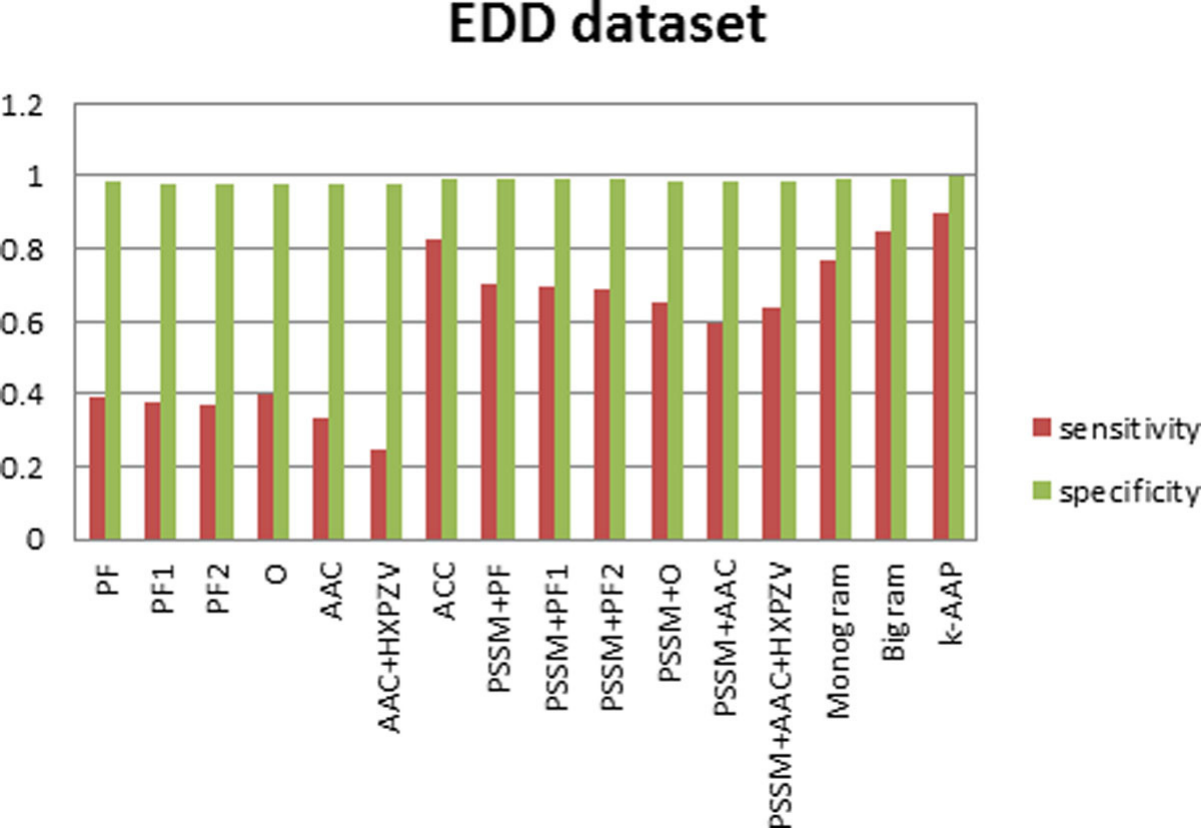
**Sensitivity and specificity of all feature sets for the EDD dataset**.

Furthermore, to assess the statistical significance, we have carried out sensitivity, specificity and precision analysis of all features used in this study, as conducted in [55]. Sensitivity is given by

$$Sensitivity=\frac{TP}{TP+FN}\times 100,$$

where TP represents true positive and FN represents false negative samples. This evaluates correctly classified test samples for each class. Specificity is given by

$$Specificity=\frac{TN}{TN+FP}\times 100,$$

where TN represents true negative and FP represents false positive. This measures correctly rejected test samples. The sensitivity and specificity values are first computed for each class, then averaged over all the classes with the results depicted in Figures 2, 3 and 4.

Furthermore, sensitivity and specificity values for all the features used here have been computed for the three datasets. Figure 2, depicts this analysis on DD dataset, Figure 3 on TG dataset and Figure 4 on EDD dataset. It can be observed from Figures 2, 3, 4 that although specificity values are is high for all the feature sets, sensitivity values are variable. This indicates that false positive is very small in comparison with true negative. Thus true negative dominates the results. This usually happens for difficult problems. It can be seen from the results that by incorporating evolutionary-based features, the sensitivity increased. This highlights the impact of evolutionary-based features in improving protein fold recognition accuracy. For all the datasets, sensitivity is highest for *k*-AAP method.

## Conclusion

In this paper, we have proposed the *k*-amino acid pair feature extraction method. This method utilizes PSSM linear probabilities and SSPM probabilities. The accuracy of fold recognition of the proposed method was consistently better than that obtained from other similar methods.

To the best of our knowledge, we achieved over 90% and 75% prediction accuracies with sequence similarity rates less than 40% and 25%, respectively. For the EDD and TG benchmark datasets, we attained 90.6% and 77.0% prediction accuracies, which are 4.7% and 8.9%, respectively, better than the best results reported in the literature. We also observed 76.1% for the DD benchmark which is 1.9% better than other methods.

## Competing interests

The authors declare that they have no competing interests.

## Authors' contributions

AS designed and wrote the manuscript. KKP financed the project and assisted in designing experiments. JL designed and conducted the experiments. AD provided the dataset and helped in the second draft of the manuscript. All authors read and approved the final manuscript.

## Declaration

The publication costs for this article were funded by Griffith University, Australia.

This article has been published as part of *BMC Bioinformatics *Volume 15 Supplement 16, 2014: Thirteenth International Conference on Bioinformatics (InCoB2014): Bioinformatics. The full contents of the supplement are available online at http://www.biomedcentral.com/bmcbioinformatics/supplements/15/S16.
